# Effect on blood lipids and body composition of a high‐fat (MUFA) and high‐fiber diet: A case–control study

**DOI:** 10.1002/fsn3.4042

**Published:** 2024-04-26

**Authors:** Patricia Romero‐Marco, Celia Chicharro, Zoraida Verde, Francisco Miguel‐Tobal, Ana Fernández‐Araque

**Affiliations:** ^1^ Department of Nursing, Faculty of Health Sciences University of Valladolid Soria Spain; ^2^ Pharmacogenetics, Cancer Genetics, Genetic Polymorphisms and Pharmacoepidemiology, Center for Drug Safety Studies, Department of Nursing, Health Sciences, Molecular Genetics of Disease – IBGM University of Valladolid Soria Spain; ^3^ Department of Legal Medicine, Psychiatry and Pathology, Biopathology‐Toxicology Laboratory, Faculty of Medicine University Complutense of Madrid Madrid Spain; ^4^ Centro de Estudios Gregorio Marañón Fundación Ortega‐Marañón Madrid Spain; ^5^ Department of Biochemistry and Molecular Biology, Faculty of Health Sciences University of Valladolid Soria Spain; ^6^ Department of Radiology, Rehabilitation and Physiotherapy; School of Medicine of Physical Education and Sport; Faculty of Medicine University Complutense of Madrid Madrid Spain

**Keywords:** body composition, dietary fiber, dyslipidemias, fatty acids, monounsaturated, Metabolic Syndrome

## Abstract

Metabolic Syndrome (MetS) is a constellation of risk factors including abdominal obesity, high triglycerides, low HDL cholesterol (HDL‐C), elevated blood pressure, and elevated fasting glucose. In Spain, according to WHO criteria, the MetS prevalence is shown to be 32% in men and 29% in women. The role of dietary habits is one of the main therapeutic strategies for the management of MetS but the most effective dietary pattern has not been established yet. This study aimed to analyze the effect of on body composition, serum lipids, and MetS components of a high‐MUFA and high‐fiber diet (HMFD). A case–control study was performed considering 40 cohabiting women. Participants were randomly assigned to HMFD group or high mono‐unsaturated diet (HMD) group to receive one of the two proposed dietary interventions. All data (serum lipids, blood pressure, height, weight, body composition, and waist circumference) were collected fasting at baseline, 55, 98, and 132 days. The HMFD group showed higher decrease in waist circumference than in the HMD group. LDL‐C dropped in both groups. Triglycerides in the HMFD group dropped during the intervention, but once the intervention was over, they returned to baseline values. The mean systolic blood pressure was lower in HMFD group. A HMFD from a weekly consumption of processed meat (Torrezno de Soria) deeply fried in extra virgin olive oil in combination with vegetables logged in a Mediterranean diet can improve MetS risk factors in healthy overweight women.

## Graphical abstract

A case–control study demonstrates that high mono‐unsaturated fatty acid fiber diet from a weekly consumption of processed meat deeply fried in extra‐virgin olive oil in combination with vegetables logged in a Mediterranean diet can improve MetS risk factors in healthy overweight women compared to high mono‐unsaturated fatty acid diet.

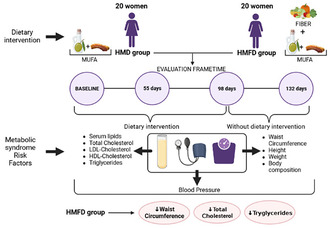

## 1 INTRODUCTION

Metabolic Syndrome (MetS) is a constellation of risk factors including abdominal obesity, high triglycerides, low HDL cholesterol (HDL‐C), elevated blood pressure, and elevated fasting glucose. MetS is associated with chronic diseases such as accelerated atherosclerotic cardiovascular disease, hyperuricemia/gout, chronic kidney disease, and obstructive sleep apnea (Cleeman, 2001).

The diagnosis of the MetS varies depending on the criteria applied, on the type, and total number of total risk factors taken into account by the different Institutions; therefore, the prevalence of the MetS might differ. Considering the latter, the National Health and Nutrition Examination Survey data estimated that 35% of adults in the United States, and as much as 50% of the over‐60 population, carry a diagnosis of MetS. Moreover, the Metabolic Syndrome and Arteries Research (MARE) (Scuteri et al., 2015) applying also the ATPIII criteria established a MetS prevalence of 23,9% in men and 24,6% in women in Europe (Scuteri et al., 2015). In Spain, according to WHO criteria, the MetS prevalence is shown to be 32% in men and 29% in women. Additionally, confinement during the COVID‐19 Pandemic has raised worldwide MetS prevalence and increased MetS risk factors due to the modification of diet and physical activity habits.

Lifestyle, particularly dietary habits, stands as the primary therapeutic strategy for the treatment and management of MetS. However, the definitive establishment of the optimal dietary pattern for managing MetS and its associated risk factors remains elusive. This uncertainty arises from the existing controversy in studies and the challenge of isolating the main beneficial nutrient, mono unsaturated fatty acids (MUFA).

In this context, the PREDIMED Study (Estruch & Emilio Ros, 2013) has demonstrated that increasing extra virgin olive oil (EVOO) intake beyond habitual levels further decreases the incidence of major cardiovascular events. The fatty acid composition of olive oil, extensively studied, reveals that it contains 60% or more of MUFA and 50%–85% of oleic acid (OA), making it the most representative food source of OA (Casaburi et al., 2013; López‐Miranda et al., 2010; Ravaut et al., 2021). Cardiovascular benefits associated with MUFA, including improved blood pressure (BP), reduced total cholesterol, elevated HDL‐C, and anti‐inflammatory effects through the inhibition of inflammatory cytokine production, are well considered (Ditano‐Vázquez et al., 2019; Marcelino et al., 2019; Schwingshackl et al., 2011; Silva Figueiredo et al., 2017; Terés et al., 2008). However, controversy persists, as some large interventions and cohort studies have reported contrasting results, albeit as the minority (Clifton, 2019). Additionally, the effects of MUFA are sometimes overshadowed by those of polyunsaturated fatty acids (PUFA), given that many studies are based on the replacement of carbohydrates with unsaturated fat (Clifton, 2019; Julibert et al., 2019). Despite these considerations, it seems logical to posit that the most beneficial replacement would involve substituting saturated fatty acids (SFA) with MUFA, although this area may not have been extensively studied (Maki et al., 2021). Moreover, available evidence supports the view that replacing SFA with unsaturated fatty acids may reduce atherosclerotic cardiovascular disease risk (Ros et al., 2015). Consequently, the food industry and the European Food Safety Authority have developed products and health claims to reach recommended intakes. In that sense, the health claim related to fatty acid intake states, “Replacing saturated fats (SFA) with unsaturated fats in the diet contributes to the maintenance of normal blood cholesterol levels (Oleic acid is an unsaturated fat)” (COMISIÓN R (UE) N o 432/2012 D LA, 2012).

Frying in EVOO may be a suitable method for successfully replacing SFA with MUFA, as several benefits have been evidenced from frying in EVOO, particularly related to antioxidant and mono‐unsaturated fatty acid transmission from the frying oil to the fried food (Ambra et al., 2022).

Furthermore, increasing dietary fiber may lower the prevalence of MetS by controlling risk factors, such as lower total cholesterol (TC), increased HDL‐C, reduced LDL‐cholesterol (LDL‐C), and BP (Fonseca Wald et al., 2014; Galisteo et al., 2008; Jang et al., 2021; Lattimer & Haub, 2010; Liu et al., 2021; Wang et al., 2022).

However, the effects of a diet rich in MUFA by the replacement of SFA and rich in fiber remain unknown, although it may be reasonable to think that a diet combining both principal nutrients will improve the totality of MetS risk factors by complementing their effects.

The hypothesis of this work is whether a combined diet rich in MUFA and Fiber would improve more the total MetS risk factors linked to body composition, serum lipids, and MetS components than a diet‐only enrichment in MUFA.

In light of the above, the aim of this study is to analyze the effect on body composition, serum lipids, and MetS components of a high MUFA and high‐fiber diet.

## 2 METHODS

### 2.1 Selection of cases and controls

A case–control trial has been carried out in a group of 40 cohabiting women aged from 18 to 90 years old. The sample size was calculated 5% significant level, *d* = 3 and S52. From this, the minimum total sample size for this study was calculated to be 38 (19 for each group). Participants were randomly assigned in a 1:1 ratio to receive one of the two proposed dietary interventions for a 98‐day period. The inclusion criteria were to be a woman and volunteer. Exclusion criteria were diagnosis of dementia and/or difficulty in swallowing.

### 2.2 Ethics

The study protocol was reviewed and approved by the BPC (CPMP/ICH/135/95) and the Ethics and Clinical Investigation Committee of the University Clinical Hospital of Valladolid (Spain). Eligible participants were informed about the study and written informed consent was attained before study participation.

### 2.3 Dietary intervention

Participants were divided into two groups: the HMD group (control group) and the HMFD group (case group). The HMFD consumed 150 g of processed meat (Torrezno de Soria) deeply fried in EVOO twice a week accompanied by 200 g of vegetables within 14 weeks while the HMD ate the 150 g of processed meat (Torrezno de Soria) deeply fried in EVOO accompanied by the regular menu. The rest of the diet was similar in both groups.

#### 2.3.1 Diet and physical exercise assessment

The diet assessment involved conducting dietary interviews with the Mother Superior at the beginning, middle, and end of the intervention. Given the nature of the center, all participants adhered to a uniform diet with standardized portions established by the Mother Superior, maintaining consistency throughout. Daily main meals comprised pulses 3 days a week, fish 2 days, and meat 2 days. Desserts accompanying the main meals included seasonal fruit and yogurt on 2 days. Participants abstained from consuming processed foods, snacks, or cakes.

As for physical exercise, participants were observed not to engage in or initiate any physical activity or exercise that could increase their usual energy expenditure. Additionally, they were asked whether, during the study period, they had incorporated or introduced any physical activity or workshop that would elevate energy expenditure compared to their routine, and no such occurrences were reported. In the interview with the Mother Superior, it was explained that their daily activities primarily revolve around maintenance work in their institution. Despite being inmates, the participants have the habit of waking up at 6 a.m. and are assigned tasks such as home maintenance, cooking, laundry, participating in a workshop where half of them work, tending to a vegetable garden, engaging in gardening, and entertainment activities, as well as sewing. Although aerobic exercise is not included, they are not considered sedentary.

### 2.4 Anthropometric and blood pressure measurements

All data were collected fasting at baseline, 55, 98, and 132 days. Height and weight were collected with bare feet and in light clothes. Height was measured to ±0.5 cm with a wall‐mounted stadiometer (HR‐200; Tanita). Body composition was analyzed by bioimpedance analysis with a Tanita DC 240 MA body analyzer. BIA measurements were performed according to the manufacturer's instructions; participants were instructed to refrain from strenuous exercise, alcohol, and caffeine intake for the 24 h before the experiment, finish their last meal at least 4 h before the measurement, and empty their bladder within 30 min before the measurement. Waist circumference was measured with a nonstretch tape halfway between the last rib and the iliac crest to the nearest millimeter by a trained nutritionist. Systolic and diastolic blood pressure was also collected with an OMRON BP7200 Upper arm blood pressure monitor.

### 2.5 Blood collection and analysis

Serum lipids were taken in fasting status from arterial blood by trained nurses. Total cholesterol, HDL‐cholesterol, LDL‐cholesterol, and triglycerides were analyzed.

### 2.6 Statistical analysis

The Statistical Package for Social Sciences software version 26.0 was used for the statistical analysis. Baseline characteristics were evaluated with descriptive analysis, including means and standard deviations. Chi‐square and independent t‐test were performed to evaluate homogeneity of the two groups. Paired‐t test was performed to evaluate the differences among data from baseline to 55, 98, and 132 days for each intervention group. Repeated measurement ANOVA (unadjusted and adjusted for age and baseline body mass index [BMI]) was applied to evaluate differences in components of MetS between the HMFD and HMD groups. Prior to analysis, the data were inspected for multivariate normality and linearity.

## 3 RESULTS

Forty participants were finally enrolled in the study. Two participants were missing the body analyzer because they were wearing stockings. Two participants dropped out due to moving to a different city, one before the third evaluation point and the other one before the fourth. Baseline characteristics of the two groups are shown in Table 1.

**TABLE 1:** Baseline characteristics of women according to the treatment group.

	HMFD			HMD			*p*‐Value
	*n*	Mean	SD	*n*	Mean	SD	
AGE (years)	20	66.25	14.68	20	31.1	10.37	<.001
BMI	21	28.03	4.93	20	24.393	2.45	<.005
FAT (%)	19	33.474	7.46	20	29.065	4.30	<.029
FFM (%)	19	66.526	7.44	20	70.95	4.30	<.028
WC (cm)	20	85.255	13.71	20	73.38	4.75	<.001
DBP (mmHg)	20	72.65	9.14	20	70	7.36	<.319
SBP (mmHg)	20	118.9	14.80	20	113.5	11.83	<.210
HR	20	75.3	13.03	20	67.1	6.36	<.016
CHOL (mg/dL)	21	206.99	27.67	20	178.675	24.26	<.001
HDL (mg/dL)	21	66.1381	9.14	20	64.685	11.58	<.657
LDL (mg/dL)	21	124.523	30.29	20	103.85	26.42	<.025
TG (mg/dL)	21	81.861	18.74	20	53.27	11.43	<.001

*Note*: Data are presented as mean ± SD; the *p*‐value was calculated with Student's *t*‐test.

Abbreviations: BMI, body mass index; CHOL, total cholesterol; DBP, diastolic blood pressure; HDL, high‐density lipoprotein; HR, heart rate; LDL, low‐density lipoprotein; SBP, systolic blood pressure; TG, triglycerides; WC, waist circumference.

### 3.1 Anthropometrical and body composition measurements

BMI has remained stable throughout the intervention in both groups. Only a little increase from baseline to 132 days has been observed being +0.58 and 0.32 in HMFD and HMD, respectively (Table 2). There have not been significant differences between groups. Similar results have been obtained for FAT% AND FFM%. The HMD group showed small differences in the FAT% and FFM% increasing 2.01 and decreasing 2.03 respectively, from Day 132 to baseline.

**TABLE 2:** Changes in mean anthropometrical and blood lipid measurements between HMFD and HMD.

Measurement	HMFD				HMD			
	Baseline	55D	98D	132D	Baseline	55D	98D	132D
	Mean ± SD	Mean ± SD	Mean ± SD	Mean ± SD	Mean ± SD	Mean ± SD	Mean ± SD	Mean ± SD
BMI	27.81 ± 4.74^a^	27.55 ± 5.09	27.89 ± 4.83	28.39 ± 4.83^a^	24.39 ± 2.44^a^	22.83 ± 5.33	24.43 ± 2.48	24.71 ± 2.55^a^
FAT (%)	33.20 ± 7.55	32.36 ± 6.97	33.66 ± 7.27	34.48 ± 6.57	29.06 ± 4.30^a,b^	28.49 ± 4.11	29.78 ± 4.55^a^	31.07 ± 4.36^b^
FFM (%)	66.80 ± 7.53	67.61 ± 6.98	66.35 ± 7.30	65.53 ± 6.60	70.95 ± 4.29^a,b^	71.51 ± 4.12	70.27 ± 4.47^a^	68.92 ± 4.38^b^
WC (cm)	82.26 ± 12.57^a,b^	80.38 ± 13.34^a^	80.65 ± 12.15^b^	82.10 ± 11.83	73.38 ± 4.75^a,b^	72.66 ± 3.80	74.17 ± 4.27^a^	74.37 ± 4.96^b^
HR (bpm)	75.37 ± 13.38^a^	65.68 ± 7.54^a^	66.95 ± 11.86	65.84 ± 7.44	67.10 ± 6.35^a,b^	73.05 ± 13.16^a^	70 ± 9.83	71.40 ± 9.17^b^
CHOL (mg/dL)	205.4 ± 26.81^a,b^	188.33 ± 23.93^a^	191.97 ± 21.29^b1^	196.56 ± 22.69^2^	178.85 ± 24.91^a^	172.35 ± 26.87^a^	172.93 ± 31.42^1^	172.39 ± 32.15^2^
HDL (mg/dL)	66.09 ± 9.44^a,b^	61.04 ± 8.81^a^	63.17 ± 10.52^b^ *	65.38 ± 8.97	64.48 ± 11.86^a,b,c^	59.99 ± 11.12^a^	59.91 ± 12.62^b^ *	60.73 ± 11.51^c^
LDL (mg/dL)	122.94 ± 6.66^a,b^	111.94 ± 5.59^a^	113.78 ± 5.06^b^	113.84 ± 5.41	104.26 ± 27.07^a^	100.26 ± 26.47	101.68 ± 31.56	97.42 ± 29.88^a^
TG (mg/dL)	82.16 ± 19.72^a^	76.51 ± 30.01	75.16 ± 20.34^a^	86.23 ± 31.28	53.32 ± 11.74^a,b^	60.76 ± 16.39^a^	56.94 ± 16.26	71.29 ± 39.27^b^
SBP (mmHg)	119.42 ± 15.01	122.84 ± 16.59^1^	118.53 ± 15.05^2^ *	122.53 ± 14.76^3^	113.50 ± 11.83	112.80 ± 10.46^1^	109.90 ± 7.41^2^ *	112.65 ± 10.19^3^
DBP (mmHg)	73.21 ± 9.034	73.58 ± 7.89	69.21 ± 7.05	67.95 ± 6.041	70 ± 7.35	74.75 ± 9.06	67.25 ± 7.60	66.75 ± 7.34

*Note*: Within‐group values with the same letter superscript (^a,b,c^) are statistically different (*p* < .05) by paired Student's *t*‐test. Between‐group values with the same number superscript (^1,2,3^) are statistically different (*p* < .05) by ANOVA‐RM unadjusted.

Abbreviations: BMI, body mass index; CHOL, total cholesterol; DBP, diastolic blood pressure; FFM%, fat‐free mass; HDL, high‐density lipoprotein; HR, heart rate; LDL, low‐density lipoprotein; SBP, systolic blood pressure; TG, triglycerides; WC, waist circumference.

* Between groups are statistically different (*p* < .05) by ANOVA‐RM adjusted by age and baseline BMI.

Heart rate (HR) underwent a significant decrease of −9.69 bpm in the HMFD group at 55 days, which was subsequently maintained across the whole intervention and up to point 132 days. Although the HMD group showed an opposite trend increasing by almost 6 bpm significantly, the differences between both groups were not significant in the ANOVA model.

The results obtained from the between‐group analysis of the WC measurement were opposite but not significant. So, while the HMFD group decreased by almost 2 cm in the mean WC, the HMD group increased by 1 cm.

### 3.2 Blood lipid measurements

**FIGURE 1:**
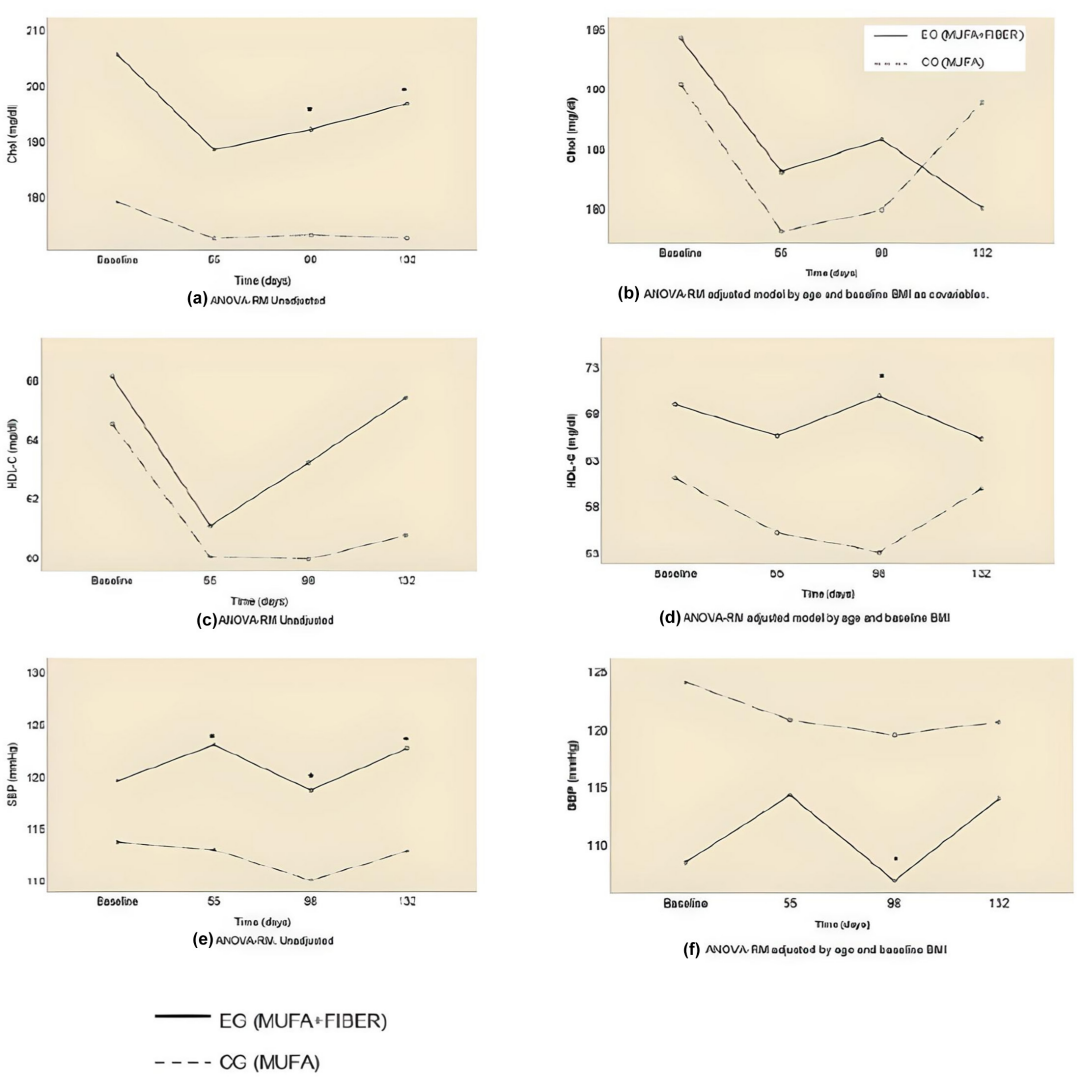
Change in Chol, HDL‐C, and SBP from baseline to 132 days in HMFD and HMD. This figure shows between‐group comparisons from ANOVA‐RM unadjusted (a,c,e) and adjusted (b,d,f) model by age and baseline BMI. BMI, body mass index; HDL‐C, HDL cholesterol; HMD, high mono‐unsaturated diet; HMFD, high‐MUFA and high‐fiber diet; SBP, systolic blood pressure.

TC decreased in both groups; however, utter reduction was greater in the HMFD group than in the HMD group. The highest fall was at 55‐day evaluation point when in HMFD group the cholesterol dropped by 8.31%, while in the HMD group, it dropped by 3.63%. Statistical differences have been observed in the within‐group analysis but not in the ANOVA‐RM adjusted model for the comparisons between groups. Despite the latter, it is interesting to observe the behavior of cholesterol in the adjusted model (Figure 1a,b), whereas the cholesterol in the HMD group rises from the end of the intervention (98 days) to the 132 days in the HMFD continues decreasing.

The most noteworthy evidence in relation to cholesterol fractions is that HDL‐C in the HMFD group experienced a statistically significant increase compared to the HMD group, which decreased. LDL‐C dropped in both groups despite the fact that in the HMFD group, the reduction was bigger than in HMD group being −9.16 mg/dL and 2.58 mg/dL, respectively, at the end of the intervention (98 days).

In line with the other blood lipid measurements, TG decreased in both groups from baseline to Day 98. Although the TG reduction was not linear in the HMD group as, at the evaluation point after 55 days, mean TG sustained a significant increase of 7.44. mg/dL. The HMFD group achieved a nonsignificant drop of 7 mg/dL at the same testing point. Worth noting is that from the end of the intervention (Day 98) to the final evaluation (Day 132) both groups raised the mean TG over baseline with an increase of 4.07 in HMFD group and 17.97 in HMD group being this hike statistically significant among HMD group.

BP, SBP as well as DBP decreased from the baseline to the end (98 days) of the intervention. However, trends in SBP change notably when adjusting the model by age and baseline BMI. So, once the model has been adjusted, the mean SBP is lower in the HMFD group but it does spike. The decreasing slope presented by the SBP in the HMD group, despite being higher than the average, is stable, linear, and continues (Figure 1e,f).

## 4 DISCUSSION

Our primary aim was to examine changes in body composition and blood lipids after consuming a HMFD by replacing SFA and adding a plateful of vegetables to a Mediterranean healthy woman. Even though participants were randomized, statistical differences were found between HMFD and HMD groups at baseline. So, to compare changes between groups, the ANOVA model was adjusted. Changes within groups are well established.

### 4.1 Effects of a HMD and HMD on anthropometric measurements

In relation to anthropometric measurements, the results of this study suggested that among the HMFD group, the BMI remained with barely any changes during the 14 weeks of the intervention, but once it was finished, the BMI increased considerably. Waist circumference decreased remarkably while HMFD participants were following the intervention patterns; however, from the endpoint up to the 132 days, it raised again to baseline values. These results occurred only in the HMFD. Differently, BMI and WC in the control group presented a little increase during the intervention and up to the final evaluation point. Therefore, due to the fact that very small effect on weight reduction on diets replacing SFA with MUFA (Bell & Culp, 2022) has been shown and that fiber has been widely studied for its role in weight management, weight loss, and adherence to diet (Maziarz et al., 2017; Miketinas et al., 2019), we might signify the beneficial function of fiber. One explanation mechanism could be due to the effect of fiber in promoting satiety and satiation. However, these effects appear to vary between different types of fiber, for example, viscous fibers were found to reduce appetite and energy intake more frequently than less viscous fibers. This is because soluble type fiber produces certain metabolites by bacterial fermentation in the gastrointestinal tract and short‐chain fatty acids (SCFA), which interact with leptin (anorexigenic hormone), which induces satiety and may be related to a decrease in food intake (Maziarz et al., 2017).

### 4.2 Effects of a HMD and HMFD on blood lipids and BP

TC decreased in both groups; however, the drop not only was higher among the HMFD than the HMD but also in the adjusted model the trends after the end of the intervention was to continue decreasing. Therefore, it seems that a diet rich in MUFA alone is not a sufficient explanation and that fiber may contribute to this behavior. The mechanisms proposed to explain the benefits of fiber would be related to its ability to limit the absorption of intestinal cholesterol and its chelating action on bile salts. Likewise, it has been seen that propionate, after being absorbed from the colon into the portal circulation, can act by inhibiting HMG‐CoA reductase, thus decreasing the endogenous synthesis of cholesterol (Finardi, 2005).

Consistent with Hooper et al (Bell & Culp, 2022) who established that replacing SFA for MUFA there was little or no effect on HDL‐C, BP, and LDL‐C, our results show that within groups HDL‐C barely decreased in both groups but with a significant but small rebound at the 98‐day evaluation in HMFD group compared to control. This difference might be explained because HMD group increased TG during the intervention and it is well known that hypertriglyceridemia promotes the exchange of TG from very low‐density lipoprotein for cholesterol esters from LDL and HDL particles, creating small, lipid‐poor particles. Small HDL particles are more susceptible to degradation, thus contributing to the low‐HDL‐C concentrations often observed in individuals with obesity or overweight (Hannon et al., 2019). Moreover, dietary fiber consumption has been hypothesized to reverse low LDL‐C and high HDL‐C through microbial fermentation and the subsequent production of SCFA, which improves glucose and lipid parameters in individuals who harbor diseases associated with dysfunctional metabolism (Cronin et al., 2021). Our results are in line with the latter because it is observed that LDL‐C in the HMFD dropped notably at the end of the intervention compared with the HMD which did not.

The impact of the combination of a diet rich in MUFA and fiber on triglycerides is interesting. Although the effects did not last after intervention, the drop in TG in HMFD was notable. Two mechanisms could have a place to explain this situation. First, the effects of fiber on energy metabolism, and secondly may be due satiating effects of fiber. Apparently, fiber affects the metabolizable energy of mixed diets by blunting the digestibility of protein, total fat, and certain SFA, thereby increasing fecal energy excretion. These reductions in metabolizable energy are modest but may be more pronounced when sustained over time and coupled with spontaneous reductions in food intake (Miketinas et al., 2019). In addition, dietary patterns that are higher in fiber are often lower in energy dense, nutrient‐poor foods that could contribute to excess adiposity (Hsiao et al., 2013).

Our results shed light on the prevention of all principal groups of MetS risk factors as a high MUFA and fiber diet worked on WC, TC, LDL‐C, TG, and BP. Related to MetS risk factors, the MARE Consortium, studied if there were differences in the risk factors combination between different European countries and found out that Southern Europe was more prevalent G‐B‐W (*fasting glucose‐elevated BP‐abdominal obesity*) cluster and Northern Europe was more prevalent T‐B‐W cluster (high triglycerides‐elevated BP‐abdominal obesity) (Scuteri et al., 2015).

The effects of a high MUFA and fiber diet on men and other population groups are needed to be explored. Related to this respect, some research is done in children and adolescents (Velázquez‐López et al., 2014).

A recent Cochrane review concluded that replacement of SFA for unsaturated fat in the form of MUFA and PUFA is effective in decreasing CVD risk due to hypolipidemic effects (Bell & Culp, 2022). This study goes further and points out novelty findings for MetS prevention. A high MUFA and fiber diet is presented as an effective dietary pattern, stronger than only high unsaturated diet, for decreasing MetS and improving MetS risk factors. More research is needed in this area with a larger sample to further strengthen the findings.

### 4.3 Strengths and limitations

This study has several limitations. First, the sample size and the differences between groups at baseline made the analysis difficult to compare both study groups. Another limitation includes the lack of knowledge of the characteristics of the diet of the participants due to the fact that their diet was not evaluated or they were not provided with any FFQ. Lastly, this study is conducted only on female participants.

## 5 CONCLUSIONS

In conclusion, a HMFD from a weekly consumption of 300 g of processed meat (Torrezno de Soria) deeply fried in EVOO in combination with vegetables logged in a Mediterranean diet can improve MetS risk factors by decreasing waist circumference, total cholesterol, and triglycerides in healthy overweight women. These findings occurred without changes in BMI, fat, or fat‐free Mass. However, strong conclusions cannot be made due to the small sample size and intervention group differences at baseline. The mechanisms associated with MetS risk factors in HMFD group are likely related to the effect of the combination of MUFA and fiber, but additional research is needed to reinforce the impact of HMFD versus HMD on MetS risk Factors and explore new findings among other population groups.

## AUTHOR CONTRIBUTIONS

**Patricia Romero‐Marco:** Conceptualization (equal); data curation (equal); formal analysis (equal); investigation (equal); software (equal); visualization (equal); writing – original draft (equal); writing – review and editing (equal). **Celia Chicharro:** Resources (equal); visualization (equal); writing – original draft (equal); writing – review and editing (equal). **Zoraida Verde:** Conceptualization (equal); formal analysis (equal); funding acquisition (equal); methodology (equal); project administration (equal); supervision (equal). **Francisco Miguel‐Tobal:** Resources (equal); validation (equal). **Ana Fernández‐Araque:** Conceptualization (equal); data curation (equal); funding acquisition (equal); methodology (equal); project administration (equal); supervision (equal); validation (equal).

## FUNDING INFORMATION

This work was supported by the Scientific Foundation of Caja Rural de Soria grant number CASVE‐NM‐21‐525 and The “Fabricantes de Torrezno de Soria” Association (info@torreznodesoria.com).

## CONFLICT OF INTEREST STATEMENT

The authors declared no conflicts of interest concerning the research, authorship, and/or publication of this article.

## Data Availability

The data that support the findings of this study are available from the corresponding author upon reasonable request.
